# Mortality measurement in transition: proof of principle for standardised multi-country comparisons*

**DOI:** 10.1111/j.1365-3156.2010.02601.x

**Published:** 2010-10

**Authors:** Edward Fottrell, Kathleen Kahn, Nawi Ng, Benn Sartorius, Dao Lan Huong, Hoang Van Minh, Mesganaw Fantahun, Peter Byass

**Affiliations:** 1 Department of Public Health and Clinical Medicine, Division of Epidemiology & Global Health, Umeå Centre for Global Health Research, Umeå UniversityUmeå, Sweden; 2 MRC/Wits Rural Public Health and Health Transitions Research Unit (Agincourt), School of Public Health, Faculty of Health Sciences, University of WitwatersrandJohannesburg, South Africa; 3 Purworejo Health and Demographic Surveillance Site, Gadjah Mada UniversityJogjakarta, Indonesia; 4 FilaBavi Health and Demographic Surveillance SiteHanoi, Vietnam; 5 Butajira Rural Health Programme, Department of Community Health, Addis Ababa UniversityAddis Ababa, Ethiopia

**Keywords:** verbal autopsy, epidemiologic transition, health and demographic surveillance systems, mortality, developing countries, health metrics

## Abstract

**Objective:**

To demonstrate the viability and value of comparing cause-specific mortality across four socioeconomically and culturally diverse settings using a completely standardised approach to VA interpretation.

**Methods:**

Deaths occurring between 1999 and 2004 in Butajira (Ethiopia), Agincourt (South Africa), FilaBavi (Vietnam) and Purworejo (Indonesia) health and socio-demographic surveillance sites were identified. VA interviews were successfully conducted with the caregivers of the deceased to elicit information on signs and symptoms preceding death. The information gathered was interpreted using the InterVA method to derive population cause-specific mortality fractions for each of the four settings.

**Results:**

The mortality profiles derived from 4784 deaths using InterVA illustrate the potential of the method to characterise sub-national profiles well. The derived mortality patterns illustrate four populations with plausible, markedly different disease profiles, apparently at different stages of health transition.

**Conclusions:**

Given the standardised method of VA interpretation, the observed differences in mortality cannot be because of local differences in assigning cause of death. Standardised, fit-for-purpose methods are needed to measure population health and changes in mortality patterns so that appropriate health policy and programmes can be designed, implemented and evaluated over time and place. The InterVA approach overcomes several longstanding limitations of existing methods and represents a valuable tool for health planners and researchers in resource-poor settings.

## Introduction

Epidemiological understanding of health and changing morbidity and mortality patterns globally is limited by inadequate measurement of population health status. Less than one-third of deaths worldwide are assigned a cause-of-death and this longstanding dearth of information, almost exclusively in the world's poorest countries, hinders understanding of mortality patterns and associated health challenges both within and across national boundaries (Mathers *et al.* 2005). As many of the world's poorer countries experience epidemiological transitions (Ng *et al.* 2006; Tesfaye *et al.* 2007; Berhane *et al.* 2008), emerging non-communicable diseases (NCDs) combined with old and new infectious disease epidemics (Tollman *et al.* 2008), as well as humanitarian crises (Fottrell & Byass 2009) and natural disasters add to the burden of already struggling health care systems and heighten the need for reliable health statistics.

In the absence of routine death registration, verbal autopsy (VA) methods gather information from a close caregiver about the signs and symptoms of the deceased's terminal illness, as well as lifestyle behaviours and other characteristics. This information is then used to derive probable causes of death, most commonly through independent review of the data by local physicians who try to reach consensus on cause of death (Snow & Marsh 1992; Soleman *et al.* 2006). VA is considered a useful method for cause-of-death ascertainment in otherwise data-poor settings; however, the approach is not without limitations. Non-standardised data-collection tools, varying skills of VA interviewers, reporting biases and knowledge and understanding of signs and symptoms of illness all affect the ability to gather reliable VA data (Chandramohan *et al.* 1994). Once data have been gathered, concerns over inter-observer agreement and lack of standardisation of physician review methods preclude meaningful comparisons of cause-specific mortality between regions and over time (Todd *et al.* 1994). In addition, the time that physicians must devote to assessing large numbers of VAs is far from ideal in areas with insufficient medical personnel. Such issues in interpreting VA data have been tackled with efforts culminating in the development of various algorithmic approaches based on the concept of distilling the process of physician review into standardised rules (Murray *et al.* 2007). Whilst being more transparent and repeatable, algorithmic procedures make it impossible to consider parallel possibilities of multiple causes of death and very often require specific questionnaires that are designed for specific contexts (Fottrell & Byass 2010).

InterVA (http://www.interva.net) is an approach to VA interpretation that aims to overcome the longstanding limitations of alternative methods. Applying Bayes’ theorem to derive probable causes of death from VA data, the method simultaneously adjusts the probability of each of a finite list of causes according to affirmative answers to a specified list of signs and symptoms, and calculates the likelihood of each cause. The method has been shown, within single settings, to produce comparable VA-derived cause-specific mortality fractions (CSMFs) to physician review whilst being 100% standardised – the same set of indicators, signs and symptoms will always lead to the same probable cause of death (Byass *et al.* 2003, 2006; Fantahun *et al.* 2006; Fottrell *et al.* 2007; Tensou *et al.* 2007). Application of the method in intervention and impact evaluation research in various settings has also been satisfactory (Bell & Qomariyah 2007; Bell *et al.* 2008; WHO, 2009), and a recent review of the INDEPTH network of Health and Demographic Surveillance Sites (Indepth, 2008) called for all sites to use the method for coding of causes of death because such approaches represent ‘the only viable strategy to produce timely and comparable cause-of-death statistics’ (Kinyanjui & Timaeus 2010).

Using InterVA and VA data from Health and Demographic Surveillance Sites (HDSS) in Ethiopia, South Africa, Vietnam and Indonesia, this paper compares mortality across four culturally diverse countries at different stages of socioeconomic development. To the best of our knowledge, this is the first demonstration of cause-of-death comparisons based on VA across diverse developing-country settings using a completely standardised interpretation tool. Our primary aim was to demonstrate the viability and value of making systematic comparisons of cause-specific mortality patterns across different settings. This paper does not attempt to draw definitive or representative conclusions concerning mortality patterns in the four countries that happen to be represented in this example.

## Methods

Health and socio-demographic surveillance involves the registration of populations within clearly circumscribed geographic areas and subsequent prospective follow-up through regular household surveys that systematically record all births, deaths and migrations. This approach underlies the Butajira (Ethiopia), Agincourt (South Africa), FilaBavi (Vietnam) and Purworejo (Indonesia) health and demographic surveillance sites (HDSS) included in this study, each a member of the INDEPTH network. Each of these HDSSs employ broadly similar methods which are described in detail elsewhere (Chuc & Diwan 2003; Ng 2006; Kahn *et al.* 2007; Berhane *et al.* 2008) and are summarised in Table 1. VA constitutes a routine aspect of the longitudinal health and demographic surveillance in each of the sites and attempts are made to gather VA data for every death. Despite broadly similar approaches in health and demographic surveillance operations, the specific methodologies, such as timing of interview, and the tools utilised for VA data capture differ between sites.

**Table 1:** Summary of period, populations and VA data included in the study from each of the demographic and health surveillance sites

Country	Ethiopia	South Africa	Vietnam	Indonesia
HDSS	Butajira	Agincourt	FilaBavi	Purworejo
Calendar period	2003–2004	1999–2004	1999	2000–2002
Number of deaths	367	3516	221	1982
Person-years of observation	52 964	394 480	43 444	167 895
Crude mortality rate (per 1000 p-years)	6.9	8.9	5.1	11.8
VAs included in study (% of all deaths)	351 (96%)	3380 (95%)	189 (86%)	864 (44%)
Further information	Berhane et al. (2008)	Kahn et al. (2007)	Chuc and Diwan (2003)	Ng (2006)

Selecting a relatively narrow time period for which VA data were available from each participating HDSS and which enabled as much temporal overlap between sites as possible, indicators were extracted for each death from each site's VA database and entered into a batch file for processing in InterVA using Microsoft FoxPro version 9 software. The process of preparing data for InterVA is straightforward, with relevant indicators being entered into a single spreadsheet format. This can be performed manually but would be time consuming for a large number of cases. Therefore, simple computer procedures were written to select the relevant information from the HDSS VA databases and transform it into the batch file, spreadsheet format used by InterVA. The process of mapping VA database variables (conceptually the same as VA questionnaire questions) to equivalent indicators in InterVA is also straightforward because the vast majority of information comes from questions that are easily associated with indicators built into InterVA.

Applying Bayes’ theorem, the computer-based InterVA approach calculates the probability of each of a finite list of causes (*C*) given the presence of specific signs, symptoms or indicators (*I*), for which the probability of reporting each indicator given a specific cause (*P*(*I*|*C*)) and the population-level probability of each cause among all deaths (*P*(*C*)) has been estimated (Byass *et al.* 2006). In mathematical terms: 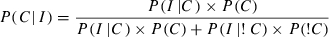 where *P*(!*C*) is the probability of not (*C*).

The prior probabilities *P*(*I*|*C*) and *P*(*C*) are derived from an expert physician consensus process whereby probabilities were estimated based on a range of 11 approximate quantitative probabilities associated with semi-qualitative descriptors that included ‘absolutely never’ (*P* = 0), ‘virtually never’ (*P* = 0.001), ‘uncommon’ (*P* = 0.002, *P* = 0.005, *P* = 0.01), ‘moderately often’ (*P* = 0.02, *P* = 0.05, *P* = 0.10), ‘frequently’ (*P* = 0.20, *P* = 0.50) and ‘almost always’ (*P* = 0.99). Thus, each step increase on the scale resulted in an approximate doubling of the probability. The physicians involved in this process were selected from a range of settings and clinical backgrounds, thus minimising the risk of developing InterVA based too closely to any one geographical region or medical discipline (Byass *et al.* 2006).

Using the above equation, the probability of occurrence of each indicator (*I*_1_…*I*_*n*_) and each possible cause of death (*C*_1_…*C*_*m*_) can be determined according to a matrix of *P*((*I*_1_…*I*_*n*_)/(*C*_1_…*C*_*m*_)). The set of indicators and causes included in the model was influenced by established VA questionnaires and the expert consensus process described above, and can be viewed elsewhere (Byass *et al.* 2006; InterVA). Symptoms, histories and circumstances of death reported in either the open narrative or closed questions in VA interviews can be utilised. Probabilities in InterVA are only affected by affirmative answers to any of the indicators; therefore, negative, missing and unknown answers do not affect probabilities, making the model more readily amenable to data collected using different tools. The fact that not reporting symptoms does not reduce or eliminate the possibility of any specific diagnoses is also important in minimising any bias that may arise from the fact that physician-derived prior probabilities could overestimate the number of recognisable and reported symptoms associated with specific causes of death in the general population as opposed to clinical samples. InterVA is currently designed to display up to three likely causes of death with corresponding likelihoods and an overall certainty factor for each death. Fewer than three causes will be displayed if the probability of the third (or second) cause is less than 80% of the probability of the preceding cause. Cases with insufficient VA data to decisively alter the cause probabilities are identified by InterVA as ‘indeterminate’.

To derive population-level CSMFs, the sum of likelihoods for each cause category is divided by the sum of the likelihoods for all causes, thus splitting individual deaths between multiple causes weighted by the cause probabilities. For example, if a case is assigned two possible causes, A and B, with likelihoods of 60% and 40%, respectively, then 0.6 contributes to the overall burden of cause A category and 0.4 contributes to the cause B category. The sum of all fractions in each cause category divided by the total number of deaths represents the population CSMF. To facilitate comparisons and with expert guidance from physician-researchers, InterVA-derived causes of death were grouped into broad categories of epidemiological and public health interest, namely cause groupings for which public health interventions would be similar.

In each HDSS, verbal informed consent is obtained at household level at every update visit and community consent from civic and traditional leadership was secured at the start of surveillance activities. In addition, the surveillance-based studies in Ethiopia, South Africa, Vietnam and Indonesia have been approved by ethics committees at the Universities of Addis Ababa, Witwatersrand, Hanoi and Gadjah Mada, respectively.

## Results

**Figure 1:**
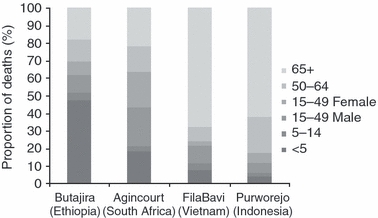
Age distributions of all-cause mortality from the four health and demographic surveillance sites.

Verbal autopsy data were available for 351 deaths from Butajira (96% of all deaths), 3380 from Agincourt (95%), 189 from FilaBavi (86%) and 864 from Purworejo (44%) between 1999 and 2004 and all were included in the study. Characteristics of the sites and the details of the VA data from each site are summarised in Table 1. Age distributions of all-cause mortality are shown by site in Figure 1.

**Figure 2:**
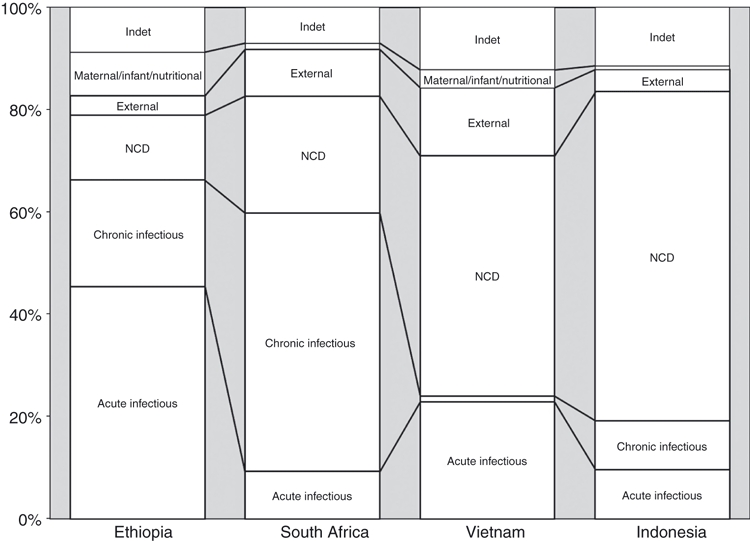
Broad groups cause classifications by country based on population cause-specific mortality fractions determined by InterVA for 4784 deaths from Butajira (Ethiopia), Agincourt (South Africa), FilaBavi (Vietnam) and Purworejo (Indonesia).

Of all VA cases, 60 (1.3%) were assigned three probable causes of death by InterVA, two causes were assigned in 592 (12.4%) cases and a single cause was assigned in 3822 (79.9%) cases. In total, 310 (6.5%) VAs provided insufficient information for cause-of-death ascertainment (ranging from 7% in Agincourt to 12% in FilaBavi), and these cases were classified by InterVA as ‘indeterminate’. Specific causes of death were grouped into broad cause classifications. Figure 2 shows the broadest groupings of cause-of-death categories for each country, indicating the major CSMF burdens in terms of infectious, non-communicable and external causes. More detail is provided in Table 2, with the categories shown in Figure 2 broken down into causes with broadly similar aetiologies, risk factors and health care implications. In Butajira (Ethiopia), the greatest burden of mortality was attributed to acute infectious causes, which include diarrhoeal diseases, malaria, meningitis, tetanus and pneumonia/sepsis. Whilst the overall burden of infectious causes of death in Agincourt (South Africa) was similar to that in Butajira, the majority of these were from chronic infectious causes of HIV-related death and pulmonary tuberculosis; acute infections represented approximately 10% of deaths in the South African data. There was also a clear burden of NCDs in this population, with approximately 20% of deaths being caused by cardiovascular diseases, chronic liver disease, respiratory diseases (excluding pneumonia), diabetes or malignancy. In FilaBavi (Vietnam), diseases of the cardiovascular system, liver disease and malignant neoplasms characterised the mortality pattern; however, more than 20% of deaths were attributed to acute infections. CSMFs for Purworejo (Indonesia) showed the greatest burden to be from NCDs, with over 60% of deaths being because of chronic conditions, predominantly cardiovascular diseases.

**Table 2:** Cause-specific mortality fractions by country as determined by InterVA for 4784 deaths from Ethiopia, South Africa, Vietnam and Indonesia

	Ethiopia	South Africa	Vietnam	Indonesia
				
Cause of death	Butajira	Agincourt	FilaBavi	Purworejo
Infection	45.4	9.2	22.9	9.5
HIV	3.4	24.5	0.0	1.2
TB	17.4	26.0	1.1	8.4
Maternal/infant	7.5	0.9	2.6	0.4
CVD	3.1	6.5	24.1	41.5
Liver	4.7	5.3	10.0	8.7
Malignancy	1.2	1.0	10.5	2.9
Diabetes	0.3	3.9	0.3	2.5
Respiratory	0.8	3.5	0.0	6.5
External causes	3.8	9.2	13.2	4.3
Other	3.5	2.8	3.0	2.5
Indeterminate	8.9	7.0	12.3	11.5

The ‘maternal/infant’ cause category consists of deaths identified as perinatal asphyxia, congenital malformations or pre-term delivery as well as adult female deaths likely to be related to pregnancy. The ‘other’ category presented in the results represents diagnoses of diseases of the nervous system, haemoglobinopathies, kwashiorkor and other malnutrition-related deaths, and digestive and urinary diseases not classified in any other category. The overall CSMF of these ‘other’ causes was similar in all settings.

**Figure 3:**
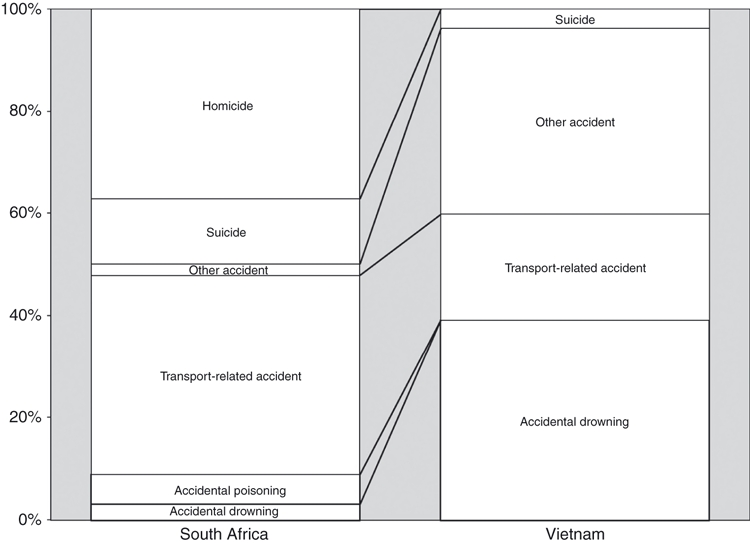
Breakdown of external causes of death in Agincourt (South Africa) and FilaBavi (Vietnam).

The burdens of external causes of deaths (which include accidental deaths, road traffic injuries, homicide and suicide) were particularly prominent in Agincourt and FilaBavi. However, InterVA's ability to differentiate between fairly specific causes revealed markedly different precise causes that go some way to characterising the different settings (Figure 3).

## Discussion

Through a standardised approach to VA interpretation, this study provides a uniquely objective view of mortality patterns in rural areas of four geographically and socioeconomically diverse countries. Although the choice of countries in this study was pragmatic, calendar periods differ between settings and the data are not necessarily representative of the countries or regions from which they come, the results demonstrate the potential of standardised methods of VA assessment for providing useful insights into health profiles on both a local and global level.

VA is generally considered a blunt tool for measuring mortality at the individual level; however, the InterVA-derived CSMFs in this study demonstrate the potential of the method to characterise sub-national profiles well. It is not the intention of our study to describe in detail the epidemiology and explanations for the different mortality profiles seen in each of the study settings. Any thorough epidemiological evaluation and discussion of the differences between settings would require careful selection of time periods and a detailed discussion of how variations in data capture processes might explain some of the differences in cause-specific mortality observed. Nevertheless, the mortality patterns identified using InterVA are plausible, illustrating populations with markedly different mortality profiles and apparently at different stages of health transition.

The breakdown of external causes in Agincourt and FilaBavi is also highly plausible (Figure 3). The external causes for FilaBavi are predominately comprised of accidental deaths, most commonly home and work-related accidents and drowning in children under 15 years (results not shown), reflecting the rural, agricultural setting and the hazardous paddy fields that characterise the physical environment (Hang *et al.* 2004). The breakdown of external causes in Agincourt shows quite a different pattern, where, according to InterVA, 40% of these deaths are a result of violence; a figure that is similar to previously reported estimates (Kahn *et al.* 1999). The distinct age-patterns of all-cause mortality between settings (Figure 1) may explain the differences in cause patterns observed to some extent as certain causes are more common in certain age groups. Derivation of age- and/or sex-specific mortality profiles, or indeed stratification by any parameter of interest, can easily be done using InterVA providing the necessary data are available. Such comparisons of InterVA-derived causes for adult female deaths in Burkina Faso and Indonesia showed distinct patterns despite underlying comparability of age and reproductive health risks (Byass *et al.* 2009). This demonstrates that the InterVA method does not simply rely on pre-judgement of causes based on underlying demographics but rather applies Bayesian principles to a whole range of indicators to derive causes on a case-by-case basis.

Whilst standardised interpretation of VA data is a major strength of this study, it must be acknowledged that, although similar, specific field procedures and tools used to gather VA data were not standardised between the four settings and none were specifically related to the InterVA method. As such not all indicators available in each country's data were built into the probabilistic model and specific indicators that are built into the model were not gathered in all settings. InterVA was intentionally designed not to relate specifically to any particular setting or questionnaire because it is important to accommodate data from different sources. Nevertheless, a need to refocus methodological developments on the entire VA process, including both data capture and interpretation may be appropriate. Recent efforts by the INDEPTH network, as well as WHO, to produce standardised and freely available VA data-collection instruments (Indepth, 2006; Baiden *et al.* 2007) are useful in this respect, but very little is known about how questionnaire design and data completeness affect the reliability and utility of VA data, whichever method of interpretation is used. Empirical research into these issues alongside developments in interpretation methods may offer new opportunities for improving cause-specific mortality data globally.

The process of mapping and translating VA database variables to equivalent indicators in InterVA is straightforward. Nevertheless, variability in questionnaires and local interpretations of medical terms requires some local input and interpretation to ensure correct mapping, particularly if information from open-ended sections is to be included. Standardisation of questionnaires would further minimise possibilities for variability in the mapping and translation process, and further research is needed to establish the value of open-ended information for computer interpretation programs. Once mapping has been established, simple computer queries can be written to fully automate the transformation of information from VA databases into indicators for InterVA. This provides opportunities for highly efficient routine procedures for timely, standardised cause-of-death ascertainment from VA data, as recommended for all INDEPTH health and demographic surveillance sites (Kinyanjui & Timaeus 2010).

Validation of the InterVA method is difficult to achieve. Because of the biases and limitations of reference standards against which cause-of-death diagnoses can be compared, validation of any VA method against medical, hospital-based data ultimately fails (Kaufman *et al.* 1997; Fantahun *et al.* 2006; Setel *et al.* 2006) and absolute gold-standard assessments of cause-of-death that can be applied to deaths that occur at home are unlikely to exist (Quigley *et al.* 1999). Nevertheless, VA diagnoses correlated with reference diagnoses continue to be considered useful in illuminating some of the limitations and misclassification errors in VA. As such, further opportunities for evaluating various aspects of InterVA do exist, including comparisons with mortality profiles derived for population sub-samples who have had contact with health facilities. Nevertheless, to consider such comparisons as validation studies would be misleading and ultimately unproductive. The optimal utility and application of VA must be judged within a broader conceptualisation of cause of death that recognises public health needs rather than perceived needs to satisfy traditional, clinical ideals of cause-of-death measurement and utility (Fottrell & Byass 2010). The outcome of such comparisons must be interpreted carefully and, rather than validity, focus on plausibility, appropriateness and usefulness in filling important gaps in mortality data (Kaufman *et al.* 1997; Setel *et al.* 2006, 2006; Fottrell & Byass 2010). In this sense, InterVA has been shown to have good comparability to physician review and offers an efficient and practical tool for cause-specific mortality assessments (Byass *et al.* 2003, 2006; Fantahun *et al.* 2006; Tensou *et al.* 2007). Still, there remain opportunities for cause-of-death misclassification, not least for HIV and TB (Tollman *et al.* 2008), which is why these causes were combined in the current analysis. It should be noted, however, that the composition of the HIV/TB cause group differs considerable between the four sites. Substantially, fewer TB deaths in Vietnam and Indonesia are related to HIV compared to Ethiopia and (in particular) South Africa, and this was reflected in the InterVA output (results not shown).

Grouping of causes of death into broad categories is useful for representing major cause-of-death burdens in relation to causation and opportunities for intervention. Consolidating causes, with multiple, related causes falling into a single category is consistent with the philosophy of focusing on cause categories and cause definitions of public health importance and broad care needs rather than traditional clinical and pathology approaches (Pacque-Margolis *et al.* 1990; Bang & Bang 1992; Marsh *et al.* 1995; Quigley 2005; Thatte *et al.* 2009). Reports of selective interventions reducing deaths to non-targeted diseases further support the use of consolidated cause categories (Marsh *et al.* 1995). Nevertheless, characterising certain causes is not always straightforward. For example, pneumonia could be characterised as a respiratory illness [as is done in the International Classification of Diseases version 10 (WHO, 2006)] but in this analysis is characterised as an infectious disease, partly because of difficulties of distinguishing between pneumonia and sepsis in very young children. Similarly, the model does not currently specify types of malignancy, although in cases where additional causes are assigned (such as respiratory disease), these can shed some light on the manifestation of the final illness. There is scope to overcome these difficulties by refining InterVA to differentiate further within broad cause categories as has been done for maternal deaths (Fottrell *et al.* 2007) and work is currently ongoing to improve specific diagnoses of causes of death in the neonatal period (Vergnano & Fottrell 2008).

The methodological limitations in comparing cause-specific mortality between settings using poorly standardised VA methods are major (Adjuik *et al.* 2006). Some studies have utilised expert and data-derived algorithms in an attempt to standardise interpretation (Quigley *et al.* 2000; Lopman *et al.* 2006); however, algorithmic approaches have important limitations, such as the inability to consider parallel possibilities of causes of death (Chandramohan *et al.* 1998; Setel *et al.* 2006; Fottrell & Byass 2010). Furthermore, by selecting single causes of death for each case, traditional algorithmic methods can lose vital information about co-morbid conditions, thereby distorting mortality estimates and underestimating potential gains from health interventions. InterVA overcomes these problems by weighting the importance of signs and symptoms in relation to specified causes of death and simultaneously adjusting the overall likelihood of each cause based on the reported signs and symptoms. This method may, therefore, more accurately reflect the interaction of different diseases that lead to death and may provide a more complete representation of the burden of diseases at the population level than has previously been possible. As such, this approach may represent a solution to the longstanding VA dilemma of quantifying a differential diagnosis process for deaths from common symptom complexes (Thatte *et al.* 2009). The reported probabilities and certainty factor in InterVA may help with interpretation of results, corresponding with the opinion that identifying possible causes and degrees of certainty of derived causes may be more useful than definitive answers in relation to VA data (Bang & Bang 1992).

Approximations of underlying probabilities in the InterVA model are sufficient to establish a workable model (Byass *et al.* 2006). Derived through expert consensus, these probabilities are based to some extent on the assumption that responses to each indicator are independent of all other indicators, which, strictly speaking, is flawed. Other techniques are being developed that use facility data to establish the probability of reporting specific symptoms given a specific cause (Murray *et al.* 2007; King & Lu 2008). These symptom properties then allow population and individual-level cause patterns to be determined from VA data from a second dataset from the population of interest. Ultimately, however, such methods are limited in that they depend upon the availability of high quality facility-based or valid mortality data for modelling – a highly context-dependent pre-requisite that cannot readily be met by the majority of settings in Africa and elsewhere that need to use VA methods. The extent to which results from population-level InterVA are sensitive to the prior probabilities has not been formally tested. However, given the somewhat approximate design of the scale from which the probabilities were selected, it is unlikely that variations in prior probabilities a step or two in either direction will dramatically affect population-level results. Further systematic testing is planned to test this hypothesis.

For the time-being, therefore, InterVA offers a viable tool for addressing the needs of public health decision-makers and researchers who want to characterise population health cheaply, efficiently and in a timely manner in settings where little or no prior knowledge is available and where VA data can be collected. Although the method may lack some of the subtlety of physician review, its simplicity and speed of data processing as well as the complete certainty that any observed differences in cause-specific mortality are a result of differences in recorded symptom profiles rather than differences in the training, skills and idiosyncrasies of coding physicians is a major advantage. Future developments in conceptual and methodological thinking around the issue of VA, combined with global collaborations and enhanced data sharing (as exemplified by the current work) are essential to achieving the clarity and consistency in health metrics needed to fill mortality data gaps at all levels.
